# N-glycans of Human Protein C Inhibitor: Tissue-Specific Expression and Function

**DOI:** 10.1371/journal.pone.0029011

**Published:** 2011-12-19

**Authors:** Wei Sun, Paola Grassi, Åke Engström, Sanjeewani Sooriyaarachchi, Wimal Ubhayasekera, Julius Hreinsson, Kjell Wånggren, Gary F. Clark, Anne Dell, Sophia Schedin-Weiss

**Affiliations:** 1 Department of Medical Biochemistry and Microbiology, Uppsala University, Uppsala, Sweden; 2 Division of Molecular Biosciences, Imperial College London, London, United Kingdom; 3 Department of Cell and Molecular Biology, Uppsala University, Uppsala, Sweden; 4 Department of Women's and Children's Health, Uppsala University Hospital, Uppsala, Sweden; 5 Department of Obstetrics, Gynecology and Women's Health, University of Missouri, Columbia, Missouri, United States of America; Bangor University, United Kingdom

## Abstract

Protein C inhibitor (PCI) is a serpin type of serine protease inhibitor that is found in many tissues and fluids in human, including blood plasma, seminal plasma and urine. This inhibitor displays an unusually broad protease specificity compared with other serpins. Previous studies have shown that the N-glycan(s) and the NH_2_-terminus affect some blood-related functions of PCI. In this study, we have for the first time determined the N-glycan profile of seminal plasma PCI, by mass spectrometry. The N-glycan structures differed markedly compared with those of both blood-derived and urinary PCI, providing evidence that the N-glycans of PCI are expressed in a tissue-specific manner. The most abundant structure (m/z 2592.9) had a composition of Fuc_3_Hex_5_HexNAc_4_, consistent with a core fucosylated bi-antennary glycan with terminal Lewis^x^. A major serine protease in semen, prostate specific antigen (PSA), was used to evaluate the effects of N-glycans and the NH_2_-terminus on a PCI function related to the reproductive tract. Second-order rate constants for PSA inhibition by PCI were 4.3±0.2 and 4.1±0.5 M^−1^s^−1^ for the natural full-length PCI and a form lacking six amino acids at the NH_2_-terminus, respectively, whereas these constants were 4.8±0.1 and 29±7 M^−1^s^−1^ for the corresponding PNGase F-treated forms. The 7–8-fold higher rate constants obtained when both the N-glycans and the NH_2_-terminus had been removed suggest that these structures jointly affect the rate of PSA inhibition, presumably by together hindering conformational changes of PCI required to bind to the catalytic pocket of PSA.

## Introduction

Protein C inhibitor (PCI) is a 57 kD glycoprotein that belongs to the serine protease inhibitor (Serpin) superfamily of proteins, and exists in many tissues and fluids in humans, including reproductive organs, semen, blood, urine, breast milk and skin [1], [2]. PCI found in blood originates from the liver and is capable of inhibiting several serine proteases involved in the regulation of coagulation and fibrinolysis, including activated protein C, thrombin, factor Xa, various kallikreins and plasminogen activators. Additionally, PCI has been found to have antimicrobial and antitumor properties and thus appears to be a medically interesting versatile protein [2].

PCI has been identified both in the human male and female reproductive tracts. The concentration of PCI in follicular fluid is similar to that in plasma [1], [3]. In contrast, a 40-fold higher concentration (3–4 µM) is present in the seminal plasma [1]. Seminal plasma PCI is mainly synthesized in seminal vesicles, where it undergoes glycosylation and is subsequently secreted in an active form. After ejaculation, it is inactivated by forming complexes with prostate-specific antigen (PSA) [4], [5], [6], t-PA (tissue-type plasminogen activator), u-PA (urokinase-type plasminogen activator) [7], and tissue kallikrein [8]. Although the function of PCI in seminal plasma is not yet completely understood, evidence showing that PCI plays a significant role in male fertility has been published. PCI knock-out mice appear to be healthy but males of this genotype are infertile due to abnormal spermatogenesis as the Sertoli cell barrier is destroyed [9]. In a clinical investigation, the inhibitory activities of PCI towards u-PA and t-PA were absent in two infertile patients, suggesting that formation of PCI complexes with u-PA and t-PA plays a role in fertilization in the human [10]. Given that the physiological role of PSA is the degradation of the major proteins of seminal coagula, Semenogelin(Sg)-I and Sg-II, PCI also appears to be involved in the regulation of semen liquefaction [11]. In addition, seminal plasma PCI has been found to inhibit the binding and penetration of human sperm to zona-free hamster oocytes [3], [12]. The inhibitor thus appears to be necessary for several steps in fertilization.

Plasma PCI has three N-glycosylation sites at Asn-230, Asn-243 and Asn-319 [13], [14]. We and other groups have observed that the N-glycans of PCI affect the rates of inhibition of several proteases [14], [15]. The primary structure of seminal PCI is identical to that of blood PCI [1]. The structures of the glycans attached to seminal PCI have, however, not previously been reported. It is therefore intriguing to investigate whether these glycans differ from those of blood PCI and whether differences in glycosylation affect the functions of PCI. Such information will be valuable for the future potential use of recombinant PCI forms in medical treatments. In this study, we have purified human seminal plasma PCI by immunoaffinity chromatography and subsequently identified the N-glycan structures by using matrix-assisted laser desorption ionization time of flight mass spectrometry (MALDI-TOF MS), which revealed marked differences compared with N-glycans from blood and urinary PCI. The majority of the seminal plasma PCI was either in an inactive, reactive-center-loop-(RCL)-cleaved form or in complex with PSA and, thus, could not be used for protease inhibition experiments. To investigate the effect of N-glycosylation on PCI inhibition of a protease from the reproductive tract, we therefore determined the PSA inhibition rates by active human blood PCI before and after enzymatic removal of either all N-linked glycans or the terminal sialic acids. These experiments were performed for both full-length PCI and a variant lacking the 6-amino-acid NH_2_-terminal peptide, previously found to constitute ∼18% of blood plasma PCI [14]. The results revealed that the N-glycans and the NH_2_-terminus together, but not alone, affect the rate of PSA inhibition.

## Results

### Purification of seminal plasma PCI

Human seminal plasma PCI was purified by using three consecutive chromatographic steps, two of which employed monoclonal antibody columns against native or RCL-cleaved PCI. In contrast to PCI from human blood [14], the majority of seminal plasma PCI bound to the second column, recognizing only RCL-cleaved PCI, indicating that most of the seminal plasma is RCL-cleaved, either free or in complex with proteases.

### SDS-PAGE and immunoblotting

**Figure 1:**
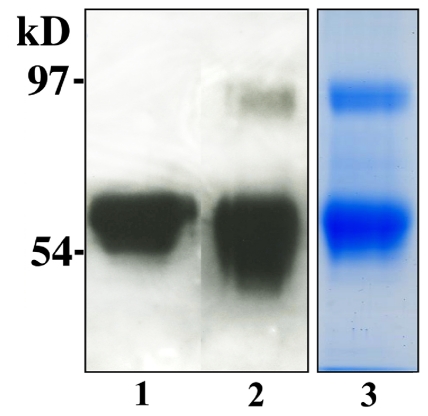
Western blot and SDS-PAGE image of purified PCI. Human seminal and blood plasma PCI were purified as described in Materials and Methods. Products were analyzed by SDS-PAGE, followed by staining with Coomassie Brilliant Blue R-250 or subjected to western blot. Lane 1, western blot analysis of blood plasma PCI; Lane 2, western blot analysis of seminal plasma PCI; Lane 3, SDS-PAGE of seminal plasma PCI.

SDS polyacrylamide gel electrophoresis (SDS-PAGE) and Western blot of the purified seminal plasma PCI revealed two broad bands, corresponding to molecular masses of 90–100 kDa and 50–60 kDa (Fig. 1), in agreement with a previous study [1]. Peptide mapping by MS revealed that the 50–60 kDa band contained cleaved PCI lacking 10 amino acids at the NH_2_-terminus and 31 amino acids at the COOH-terminus, consistent with the protein being both NH_2_-terminally cleaved and RCL-cleaved (spectra not shown). The 90–100 kD band was found to contain both PSA and cleaved PCI, verifying that a portion of PCI in seminal plasma is in an SDS-stable complex with PSA, in agreement with previous studies [1], [4]. In order to obtain pure PCI for N-glycan structure analysis, another gel chromatography step was further employed to separate the free form of PCI from the PCI-PSA complex.

### MALDI-MS analysis of N-glycans released from PCI

**Figure 2:**
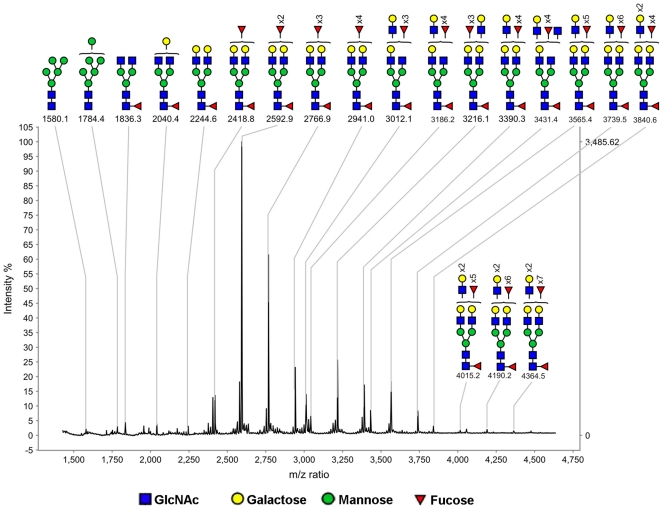
MALDI-TOF mass spectrum of permethylated PCI N-glycans from human seminal plasma. N-glycans were derived from 50% (v/v) acetonitrile fraction from a C_18_ Sep-Pak. All molecular ions are [M+Na]^+^, and nomial masses of the ^12^C isotope are shown. Putative structures based on composition and knowledge of biosynthetic pathways are shown. The symbols for the monosaccharide units are explained in the picture.

Purified PCI from human seminal plasma was reduced/carboxymethylated and tryptically digested to facilitate deglycosylation with PNGase F. The released glycans were separated from peptides and were analyzed by MALDI-MS after permethylation and Sep-Pak purification (Fig. 2). The spectrum indicates that PCI N-glycans have compositions consistent with mostly core-fucosylated bi-antennary glycans, but some tri-antennary and tetra-antennary glycans were also observed. The most abundant ion (m/z 2592.9) has a composition (Fuc_3_Hex_5_HexNAc_4_) consistent with a core fucosylated bi-antennary glycan with two terminal Lewis^x^. The second most abundant ion (m/z 2766.9) has a composition (Fuc_4_Hex_5_HexNAc_4_) consistent with a core fucosylated bi-antennary glycan with one terminal Lewis^x^ and one terminal Lewis^y^. Altogether, various combinations of Lewis^x^, Lewis^x^/Lewis^x^, Lewis^x^/Lewis^y^ and Lewis^y^/Lewis^y^ were observed on the two–four antennae. The compositions of the peaks with five or more fucose residues are consistent with core fucosylation together with Lewis^x^ and Lewis^y^ epitopes on the three or four antennae, as previously described by Pang et al [16]. The high mannose content was very low and virtually no sialylation was detected (Fig. 2 and Fig. S1).

### Preparation of blood plasma PCI

**Figure 3:**
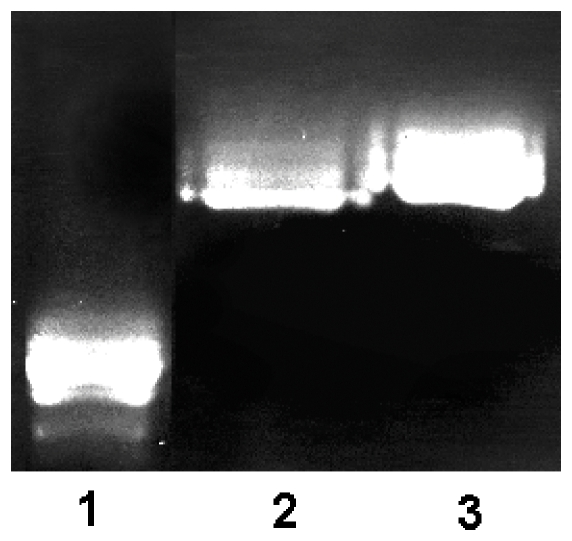
Native PAGE of desialylated blood plasma PCI. PCI was desialylated,,purified and analyzed by native PAGE, as described in Materials and Methods. The gel was stained with Ruby protein stain. Lane 1, native PCI; Lane 2, desialylated N-terminally cleaved PCI; Lane 3, desialylated full-length PCI.

PNGase F treated PCI was isolated using heparin-Sepharose chromatography with an extended NaCl and pH gradient, as described previously [14]. Desialylated PCI was isolated using ion exchange chromatography and subsequently analyzed by native PAGE (Fig. 3). Intact PCI migrated considerably further than the desialylated PCI forms, verifying that the negatively charged sialic acids had been efficiently removed by the neuraminidase treatment. In addition, desialylated NH_2_-terminally cleaved PCI (Fig. 3. Lane 2) migrated slightly further than desialylated full-length PCI (Fig. 3. Lane 3).

### Effects of PCI variants on PSA inhibition

**Figure 4:**
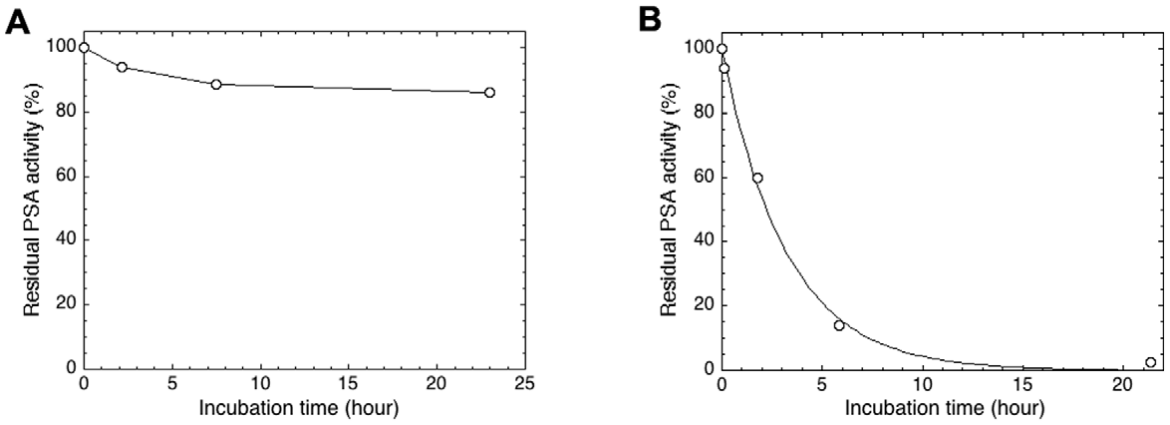
Rate of PSA inhibition by full-length native PCI derived from blood. The inhibition of PSA was measured as a function of time by a discontinuous assay, as described in Materials and Methods.

**Figure 5:**
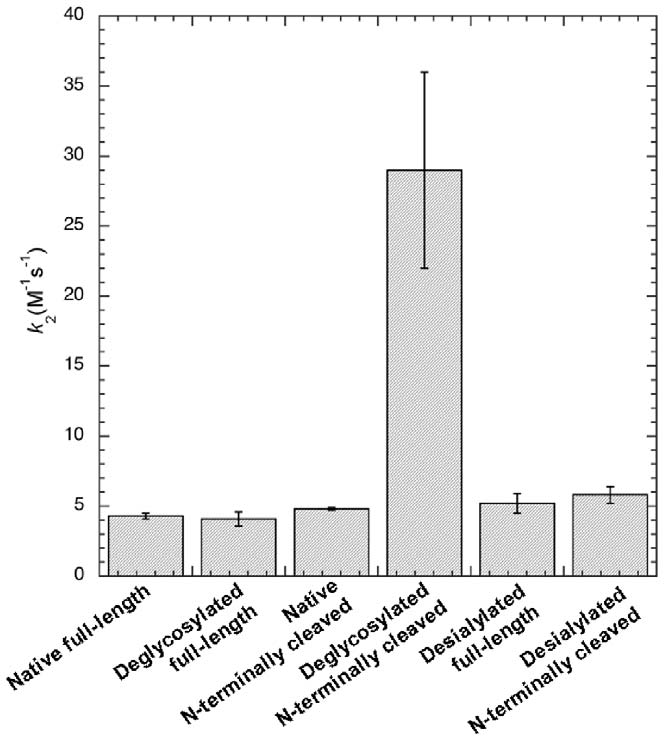
Effects of N-glycans and the Δ6-N-terminus on the rate of PCI inhibition of PSA. The inhibition of PSA by untreated full-length, untreated Δ6-N-cleaved, deglycosylated full-length, deglycosylated Δ6-N-cleaved, desialylated full-length and desialylated Δ6-N-cleaved PCI isolated from blood plasma were measured as a function of time by a discontinuous assay. The resulting second-order rate constants are displayed. Data are the mean ± SD of two independent experiments, each based on 4–6 time points.

Four PCI variants derived from human blood plasma were used to determine the effects of N-glycans and the NH_2_-terminus on the rates of PSA inhibition. The same variants were previously used for studies of inhibition of factor Xa and thrombin [17]. The stability of PSA was first evaluated over a 24-hour period in the inhibition assay conditions used (Fig. 4A), verifying that the enzyme retained 86% of the activity and is thus reasonably stable. The effects of N-glycan removal and the 6 residue NH_2_-terminal peptide of PCI on the rate of PSA inhibition were studied at 25°C. PSA inhibition of intact and PNGase F-treated PCI fitted to a monophasic single-exponential decay function (Fig. 4B). Similarly, deglycosylated PCI with and without the NH_2_-terminus also fitted to this type of function. A comparison of the second-order rate constant for PSA inhibition by the four PCI variants is shown in Fig. 5. These constants were 4.3±0.2 and 4.1±0.5 M^−1^s^−1^ for full-length PCI and the variant lacking the six NH_2_-terminal residues, respectively, showing that the rate of PSA inhibition was not affected by removing the 6 NH_2_-terminal residues. Moreover, second-order rate constants were 4.8±0.1 and 29±7 M^−1^s^−1^ for the PNGase F-treated full length and NH_2_-terminal-free PCI, respectively, demonstrating that the rate of PSA inhibition was not affected by the removal of N-glycans alone, whereas the combined loss of N-glycans and the NH_2_-terminal 6-residue peptide resulted in a 7–8 fold increase in the rate of PSA inhibition. One major difference between the N-glycans of PCI from blood and seminal plasma was the absence (seminal PCI) or presence (blood PCI) of negatively charged sialic acids. To investigate whether the joint effect of the N-glycans and the NH_2_-terminus on PSA inhibition was affected by the sialic acids, we therefore also determined the effects of neuraminidase treatment of blood PCI on the rates of PCI inhibition of PSA. Second-order rate constants were 5.2±0.7 and 5.8±0.6 M^−1^s^−1^ for the neuraminic acid free, full length and neuraminic acid free, NH_2_-ternimally cleaved PCI, demonstrating that sialylation of PCI does not have any major effect on the rate of PSA inhibition. All the rate constants are expressed as the mean ± SD of two independent experiments, each based on 4–6 time points.

### Modeling of the PCI-PSA interaction

**Figure 6:**
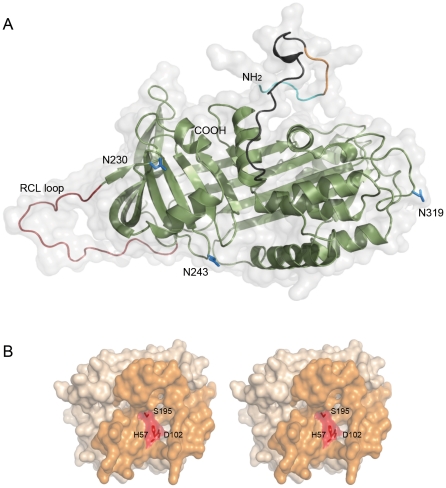
Modeling of the NH_2_-terminal parts of PCI and the PCI-PSA complex. (A) Ribbon cartoon of PCI (PDB entry 2OL2) with the fantasy model of the NH_2_-terminal part. Residues 1–6 (HRHHPR), 7–10 (EMKK) and 11–28 (R11 – D28) of the fantasy model are shown in cyan, orange and black respectively. N-glycan-attaching residues and the RCL loop, which interact with PSA in the PCI-PSA complex, are shown in blue and brick red, respectively. Amino- and carboxy-termini are labeled NH2 and COOH, respectively, where the surface of PCI is shown in light grey. (B) Stereo illustration of the surface of PSA, displaying the catalytic pocket forming loops in orange. The catalytic residues are marked in red. The RCL loop of PCI binds to this catalytic pocket.

Because there are no crystal structures available for the PCI-PSA complex or the NH_2_-terminus of PCI, we created models of these structures. The displayed conformation of the RCL loop of PCI, as present in the human PCI crystal structure (PDB entry 2OL2), does not fit well into the catalytic pocket of PSA. We therefore show the two molecules individually (Fig. 6). Adjustments are consequently required for optimal complex formation. The suggested conformational change is supported by the flexibility of the RCL loop observed in related crystals structures [18], [19].

## Discussion

Due to the versatility of PCI, the mechanisms of regulation of its various functions are intriguing to investigate. Previous studies have shown that post-translational modifications of the inhibitor, i.e. glycosylation and protease processing, affect the specificity of PCI for proteases [14], [15], [20]. However, many clues about the structure/function of the covalently linked glycans, as well as the segments of the inhibitor that are proteolytically released, remain unknown. Herein, we show for the first time the structural profile of N-glycans of human seminal plasma PCI, determined by mass spectrometric methods. Moreover, we report the effects of the N-glycans and the NH_2_-terminus on the rate of inhibition of PSA, a major serine protease in seminal plasma. Since the seminal plasma glycoforms of PCI are inactive, we used the four PCI variants derived from blood that were previously employed to study kinetics for factor Xa and thrombin inhibition. Testing of these variants allowed us to observe different effects of the N-glycans and the NH_2_-terminus on the three proteases.

Our group previously reported that blood plasma PCI is micro-heterogeneous, which was revealed by the appearance of at least six clear bands in SDS-PAGE [14]. The various PCI sizes were found to be caused by differences in N-glycan structures, N-glycan occupancy and the presence of two forms that differ by the presence or absence of six amino acids at the NH_2_-terminus. All three potential N-glycosylation sites were occupied in the majority of PCI, although a small fraction of the PCI sample lacked the glycan at Asn-243 [14]. In contrast, the SDS-PAGE of seminal plasma PCI reported here does not show any clear separation of PCI variants, although the broad appearance of the band in the gel indicates that there are several variants that are not as well separated. This difference in appearance on SDS-PAGE of blood PCI compared to seminal plasma PCI is presumably explained by the differences in posttranslational modifications. For instance, all seminal plasma PCI lacked an NH_2_-terminally cleaved peptide, although this peptide was ten residues (HRHHPREMKK) instead of six. This ten-residue NH_2_-terminal peptide is highly positively charged and thus likely affects the functional properties of PCI.

**Figure 7:**
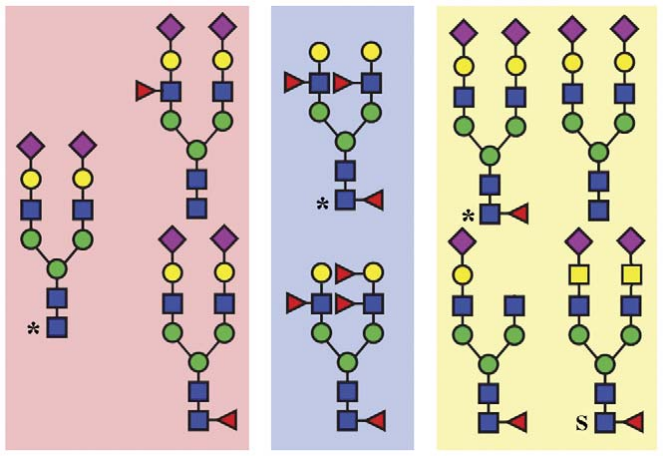
Tissue-specific expression of N-glycans on PCI. Major N-glycans of PCI derived from blood plasma, seminal plasma and urine are framed in light red, blue and yellow, respectively. N-glycan structure of urinary PCI is drawn according to [13]. * represents the most abundant glycan. Glycan marked with “S” has a special lacdiNAc (GalNAcβ1-4GlcNAc) structure (constitutes about 12% of the total N-glycans in urinary PCI [13]). This structure has not been observed in blood or seminal plasma PCI N-glycans.

The N-glycans of seminal plasma PCI consist mainly of core-fucosylated, biantennary lewis^X^ and/or lewis^Y^-capped structures. They are completely devoid of sialic acids, and therefore differ markedly in sequence from those previously identified in blood PCI [14]. Our previous study showed that the N-glycans from blood PCI consist of bi-, tri, and tetra-antennary structures of which the most abundant structure is a non-fucosylated bi-antennary glycan with both antennae capped with sialic acid [14]. A small fraction of the blood PCI N-glycans carried sialyl-Lewis^X^ epitopes. The N-glycans linked to urinary PCI consist of mainly core fucosylated, biantennary structures that are to a great extent sialylated at the end of the antennae [13]. Additionally, a portion of the urinary PCI glycans have antennae composed of lacdiNAc (Gal NAcβ1-4GlcNAc), a rarer sequence that has been observed in neither blood nor seminal plasma PCI N-glycans. The source of urinary PCI has not been completely identified so far. However, Radtke et al. have shown that PCI is synthesized in tubular cells of the kidney, suggesting that the kidney is a source for urinary PCI [21]. The differences observed in N-glycan structures of PCI in seminal plasma, urine and blood supports this conclusion and demonstrates that the N-glycosylation of PCI displays a highly tissue-specific expression (Fig. 7).

A recent study revealed the overall seminal plasma N-glycome, which consists of bi-, tri- and tetraantennary sequences [16], of which several contain lewis^X^ and/or lewis^Y^-capped structures. In contrast to the N-glycans of seminal plasma PCI, the seminal plasma N-glycome also contains a substantial portion of high-mannose as well as sialylated structures. Moreover, sialylated glycans are abundant in seminal plasma from some individuals and minor in others according to this glycomics analysis, while they appear to be totally absent in PCI. Our results thus demonstrate that PCI neither contributes to the individual differences in sialylated N-glycans nor to the high-mannose structures observed in the seminal plasma glycome.

The highest concentration of PCI in human is found in seminal plasma. Although the exact physiological role of PCI in semen is still not clear, we have reason to believe that PSA is a major target protease for PCI inactivation in the semen, because PSA occurs at a concentration of 15–60 µM, is the most abundant serine protease in seminal fluid [22] and about 34–44% of the total PCI in semen is in complex with PSA [4], [5]. Even so, rate constants for the inhibition of PSA by PCI have not previously been documented. Since all purified seminal plasma PCI was in the proteolytically modified and inactive state, blood plasma PCI was used to determine the PSA inhibition rates. The second-order rate constant (*k_2_*) for PSA inhibition by native full-length PCI and PCI lacking the 6-residue NH_2_-terminal peptide were not statistically different (4.3±0.2 and 4.1±0.5 M^−1^s^−1^, respectively). This finding indicated that the six NH_2_-terminal residues do not contribute to the PSA inhibition rates. The same rates are 2.7×10^3^ M^−1^s^−1^, <1×10^3^ M^−1^s^−1^, 2.4×10^5^ M^−1^s^−1^ for PCI inhibition of t-PA, u-PA and acrosin, respectively [14], [23], [24], [25]. The rate for PSA inhibition by PCI is thus very low compared to the inhibition of other seminal serine proteases, although a large proportion of PCI in seminal plasma forms a complex with PSA. Similar observations have been reported previously and are presumably due to the high concentration of PSA in semen [2], [5]. Moreover, N-glycans alone did not significantly contribute to the *k*_2_ for PCI inhibition of PSA. However, the combined loss of N-glycans and the Δ6-NH_2_-terminus significantly enhanced the reaction, indicating that these structures together contribute to the slow PSA-PCI reaction velocity. These results may be explained by the possibility that N-linked glycans and the NH_2_-terminus together sterically hinder a conformational change required for the RCL loop of PCI to fit into the catalytic pocket of PSA. This explanation is reasonable considering that for serpin-protease inhibition reactions it has been proposed that the first step, i.e. the formation of the encountering complex, is rate limiting [26]. Since the terminal neuraminic acid on PCI did not have any major effect on PSA inhibition rates, we further concluded that the shedding caused by the N-glycans and the NH_2_-terminus together is not affected by the charge of the N-glycans. It will be highly interesting in future investigations to determine the effects of the seminal plasma-specific posttranslational modifications on PCI functions, such as the inhibition of various proteases and cell-surface receptor interactions. However, protease inhibition experiments will require the isolation of active seminal plasma-derived PCI, which has proven to be very difficult to achieve, due to the high concentrations of serine proteases in seminal plasma. Alternatively, it may be possible to produce recombinant PCI expressing the seminal plasma PCI N-glycans, although this is a difficult task because it requires the precise expression of the correct glycosyltransferases.

A recent study indicates that PCI could also play another functional role in the human male and female reproductive systems. The immune lectin designated DC-SIGN (dendritic cell-specific intercellular adhesion molecule-3-nonintegrin) is associated with both mature and immature dendritic cells (DCs) [27], [28]. Many human pathogens bind to DC-SIGN, enabling their detection, uptake and the development of specific adaptive immune responses by DCs [29]. However, DC-SIGN also binds to several endogenous glycoproteins, and such interactions are currently thought to promote immune homeostasis [28]. Many proteins are specifically produced in the male urogenital tract after the onset of puberty, but they have not been subjected to thymic education. Such autoantigens could trigger immune responses in both the human male and female reproductive systems [30]. However, PCI and three other glycoproteins (clusterin, galectin-3 binding protein, and prostatic acid phosphatase) have recently been defined as endogenous glycoprotein ligands for DC-SIGN in seminal plasma [31]. Extensive fucosylation was important for these interactions. Therefore seminal plasma PCI could also have an immunomodulatory effect in both the male and female reproductive tracts, in which fucosylation plays a critical role.

The results presented here provide further support that posttranslational modifications affect the functional specificity of PCI, which is medically relevant because PCI can act for instance as an anti-inflammatory and antitumor agent. It is also essential for all stages of reproduction. Therefore it may be used for therapeutic purposes. It was previously shown that the overall removal of N-linked glycans and the NH_2_-terminal peptide of PCI affect the inhibition rates in the presence but not in the absence of the cofactors heparin and thrombomodulin [14]. For factor Xa, the NH_2_-terminal peptide of PCI was found to affect the inhibition rates both in the absence and the presence of heparin [14]. PSA, in contrast, is unique in that only the combination of the N-glycans and the NH_2_-terminus of PCI affect the rate of its inhibition.

## Materials and Methods

### Seminal plasma PCI purification

This study had been approved by the Regional Ethical Review Board committee in Uppsala, at Uppsala University and at the Uppsala Univerity Hospital. We obtained informed written consent from all participants involved in the study. Human semen, obtained from several healthy donors (according to World Health Organization guidelines), were allowed to liquefy and then centrifuged at 500–1000 g for 10 min. The supernatant was collected and then mixed with 10 mM (final concentration) benzamidine chloride (Sigma-Aldrich, St. Louis, US). Seminal plasma was then stored at −20 °C and the samples from several individuals were pooled before use. Purification of PCI was carried out as described before [14] with only minor changes. Briefly, three consecutive affinity chromatography steps were employed, with the use of two immobilized monoclonal antibodies and one heparin-Sepharose chromatography step. The first immobilized antibody recognizes all forms of PCI, whereas the second antibody recognizes only RCL-cleaved and complexed forms of PCI. To separate the free seminal PCI from its complex forms, an additional gel chromatographic step, using a Superdex 75 column (GE healthcare, Uppsala, Sweden), was employed. Protein was then concentrated by filter centrifugation using Amicon Ultra-4 centrifugal filter devices (Millipore Corp., Bedford, MA). Protein concentration was determined by UV absorbance with the use of the extinction coefficient determined previously at 280 nm (31300 M^−1^cm^−1^) [32].

### SDS-PAGE and immunoblotting

The purified protein samples were analyzed by SDS-PAGE in 10% gels with the Laemmli [33] system. Gels were then subjected to Coomassie Brilliant Blue R-250 staining or immunodetection. For western blot, protein bands were transferred onto a nitrocellulose sheet (Schleicher & Schuell BioScience, Dassel, Germany). A primary antibody, rabbit anti-human PCI (Abcam, Cambridge, UK) and a HRP (horseradish peroxidase) conjugated secondary antibody against rabbit (Dako, Glostrup, Denmark) were used. The signal was developed using ECL plus reagents (GE healthcare, Uppsala, Sweden) and the membrane was then exposed to Fuji film.

Bands were excised from the Coomassie stained SDS-PAGE gel; then subjected to MS analysis for peptide mapping, as described previously [14].

### MS analysis for N-glycan structure determination

Purified PCI was reduced in 600 mM Tris-HCl buffer, pH 8.4, containing 2 mg/ml dithiothreitol (37°C for 45 min), and carboxymethylated by addition of 12 mg/ml iodoacetic acid (room temperature for 1.5 h). Carboxymethylation was terminated by dialysis against 50 mM NH_4_HCO_3_, pH 8.5, at 4°C for 48 h, followed by lyophilisation. PCI was incubated with trypsin (Sigma) at a 50∶1 ratio (w/w) in 50 mM NH_4_HCO_3_, pH 8.4, for 16 h at 37°C. The digestion was terminated by heating at 100°C for 3 min, followed by C_18_ Sep-Pak chromatography (Waters Corp.). Bound peptides were eluted with either 20% (v/v) or 40% (v/v) propanol in 5% aqueous acetic acid, pooled and lyophilised. PNGase F (Roche) digestion was carried out in 50 mM ammonium bicarbonate, pH 8.5, for 16 h at 37°C with 3 Roche U of enzyme. The released N-glycans were separated from peptides by Sep-Pak C_18_ (Waters Corp.) as described [34]. Permethylation of glycans was performed using the sodium hydroxide procedure [34]. MALDI-TOF data were acquired on a Voyager-DE sSTR mass spectrometer (PerSeptive Biosystems, Framingham, MA, USA) in the reflectron mode with delayed extraction. Permethylated samples were dissolved in 10 µl of methanol in water, and 1 µl of dissolved sample was pre-mixed with 1 µl of matrix (10 mg/ml 2,5-dihydroxybenzoic acid (DHB) in 80% (v/v) aqueous methanol) before loading onto a metal plate.

### Preparation of blood plasma PCI

Blood plasma PCI used for kinetic studies was purified as described previously [14]. To obtain N-glycan free PCI, the native protein was treated with PNGase F, as described previously [14]. The native and the N-glycan-free PCI forms were further subjected to an extended heparin sepharose gradient as described previously, to separate full-length PCI from the form lacking a 6-residue NH_2_-terminal peptide [14]. Neuraminic acid-free PCI was prepared by incubating 300 µg PCI with 15000 NEB U neuraminidase (New England BioLabs) in 20 mM Tris-HCl, 0.1 M NaCl, pH 7.4 at 37 °C for 3 hours. This Neuraminidase catalyzes the hydrolysis of α2–3, α2–6, and α2–8 linked N-acetyl-neuraminic acid residues from glycoproteins and oligosaccharides. Ion exchange chromatography on a mono Q 5/50 GL column (GE Healthcare, Uppsala, Sweden) was used to separate full-length PCI from NH_2_-terminally cleaved PCI. A gradient was employed with running buffers A and B containing 0.1 and 1 M NaCl, respectively, in 20 mM sodium phosphate, 0.1 mM EDTA and pH 7.4. The gradient was segmented as follows: 0 min, 0% B; 60 min, 40% B; 65 min, 100% B; 67 min, 0% B; 72 min, 0% B. The flow-rate was 0.5 ml/min. The resulting peaks were collected and concentrated, and the buffer was exchanged into 20 mM Tris-HCl, 0.1 M NaCl, pH 7.4, with Amicon Ultra-4 centrifugal filter devices (Millipore Corp.). The protein fractions were then analyzed by 7.5% native PAGE.

### Kinetics of PSA inhibition

Second-order rate constants for the inhibition of PSA by the PCI variants were measured under pseudo-first-order conditions, using a discontinuous assay [35] in 20 mM Tris-HCl, 0.1 M NaCl and pH 7.4 at 25°C. PSA (final concentration 350 nM) was incubated with a 10-fold excess of PCI. After different reaction times, varying from 30 min to 24 hours, aliquots were diluted in the assay buffer containing 400 µM chromogenic substrate Meo-Suc-Arg-Pro-Tyr-pNA (Calbiochem, Darmstadt, Germany) in the final system. The initial rates of the residual PSA activity were monitored in a Tecan Infinite M200 96-well plate reader at 405 nm and plotted against the incubation times. Observed pseudo first-order rate constants, *k*_obs_, were obtained by nonlinear least-squares fitting of such plots to a single-exponential decay function based on 4–6 time points [35]. Second-order rate constants were calculated by dividing *k*_obs_ by the active PCI concentration [35].

To ensure that the loss of PSA activity over time in the PCI inhibition assay was caused by PCI inhibition, the stability of PSA over time was determined under the same conditions as described above. PSA (350 nM) was incubated in the assay buffer at 25 °C. At different time intervals, from 2 to 24 hours, aliquots were drawn from the incubation, and the residual protease activity was measured.

### Modeling of PCI and PSA

A fantasy model of the NH_2_-terminus (from residues His1-Asp28) of PCI was modeled using crystal structures similar to PCI. The PDB entry 2OL2 [36] was the starting model to build the additional residues in the crystallographic program O. PSI-BLAST searches [37] located the similar structures in the protein data bank (PDB) [38]. The structures were superimposed by the program LSQMAN [39] and inspected in O. The modeling of the PCI-PSA complex was accomplished by replacing the molecules of alaserpin and anionic trypsin II complex structure (PDB entry 1K9O) [40] with those of PCI (sequence identity 24%) and PSA (PDB entry 2ZCH, sequence identity 42%) [41], respectively. Figure 6 was prepared using PyMOL (http://www.pymol.org/).

## Supporting Information

Figure S1 **Predicted sialylated structures.** Cartoon representations of predicted sialylated structures are shown in the bottom panel. Structures are based on a previous study on human seminal plasma by Pang et al. (reference 16). The upper panel shows a zoomed view of human seminal plasma PCI MALDI-TOF spectrum. The light blue arrows indicate the expected m/z values of predicted sialylated structures. No peak corresponding to the expected m/z values have been observed above the background noise.
(JPG)
