# Supplementary figures and images for: N-glycans of Human Protein C Inhibitor: Tissue-Specific Expression and Function
Source: PLoS One. 2011 Dec 19;6(12):e29011. doi: 10.1371/journal.pone.0029011 (PMC3242763; doi:10.1371/journal.pone.0029011)

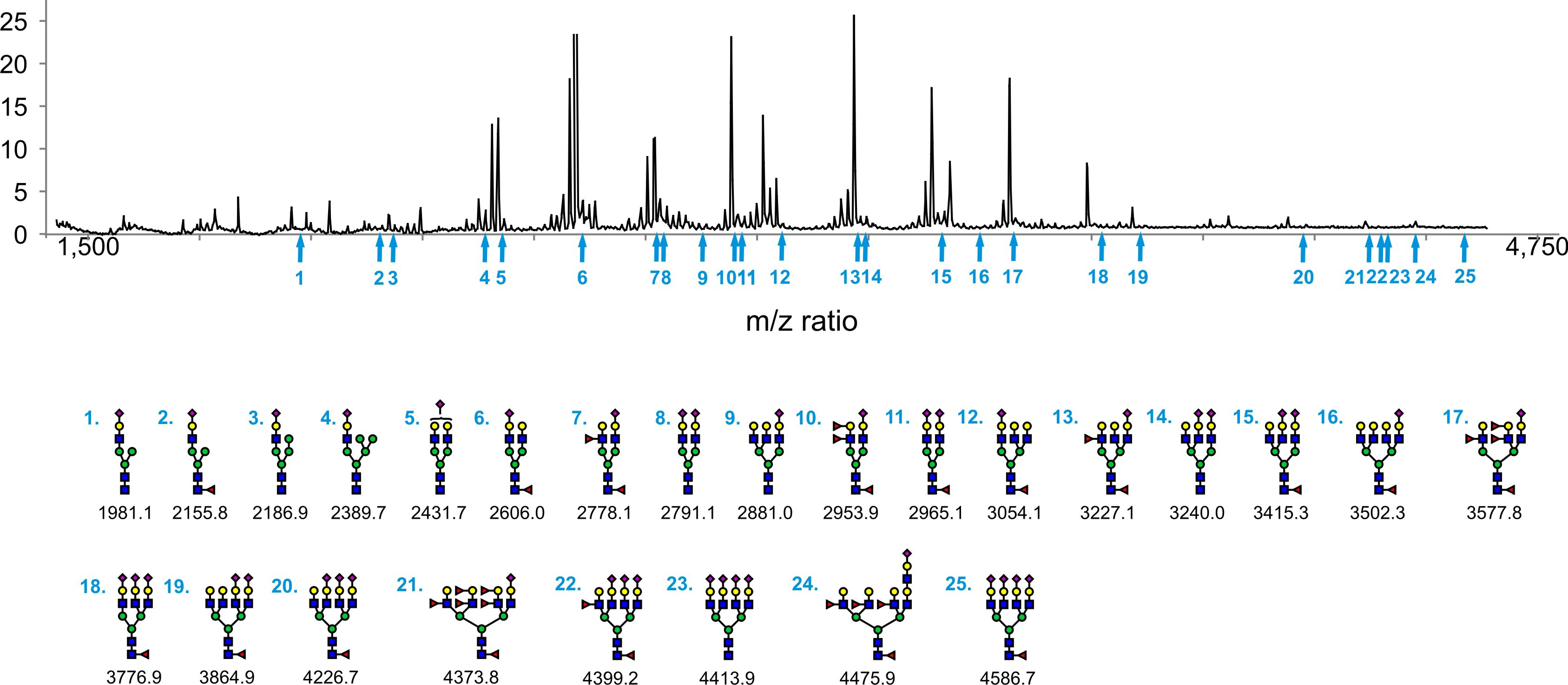

Supplement: Figure S1 — Predicted sialylated structures. Cartoon representations of predicted sialylated structures are shown in the bottom panel. Structures are based on a previous study on human seminal plasma by Pang et al. (reference 16). The upper panel shows a zoomed view of human seminal plasma PCI MALDI-TOF spectrum. The light blue arrows indicate the expected m/z values of predicted sialylated structures. No peak corresponding to the expected m/z values have been observed above the background noise. (JPG) [file pone.0029011.s001.jpg]
